# Identification and Functional Characterization of Tyrosine Decarboxylase from *Rehmannia glutinosa*

**DOI:** 10.3390/molecules27051634

**Published:** 2022-03-01

**Authors:** Yuanjun Li, Yanan Wang, Longyu Huang, Chunrong Chen, Na An, Xiaoke Zheng

**Affiliations:** 1 College of Pharmacy, Henan University of Chinese Medicine, Zhengzhou 450046, China; liyuanjun@hactcm.edu.cn (Y.L.); wangyanan4819@163.com (Y.W.); anna_8596@163.com (N.A.); 2 Institute of Cotton Research, Chinese Academy of Agricultural Sciences (CAAS), Anyang 455000, China; huanglongyu1@163.com; 3 School of Life Science, Henan University, Kaifeng 475004, China; chenchunrong1998@163.com

**Keywords:** *Rehmannia glutinosa*, acteoside, tyrosine decarboxylase

## Abstract

*Rehmannia glutinosa* is an important medicinal plant that has long been used in Chinese traditional medicine. Acteoside, one of the bioactive components from *R. glutinosa*, possessed various pharmacological activities for human health; however, the molecular mechanism of acteoside formation is not fully understood. In the current study, a novel tyrosine decarboxylase (designated as RgTyDC2) was identified from the *R. glutinosa* transcriptome. Biochemical analysis of RgTyDC2 showed RgTyDC2 uses tyrosine and dopa as the substrate to produce tyramine and dopamine, respectively, and it displays higher catalytic efficiency toward tyrosine than dopa. Moreover, the transcript level of *RgTyDC2* was consistent with the accumulation pattern of acteoside in *R. glutinosa*, supporting its possible role in the biosynthesis of acteoside in vivo.

## 1. Introduction

*Rehmannia glutinosa* Libosch. (Dihuang), a medicinal herb from the Scrophulariaceae family was widely used in traditional Chinese medicine for thousands of years. *R. glutinosa* showed positive effects on the blood system, immune system, cardiovascular system, nervous system and of being anti-tumor, etc [1,2,3,4,5,6,7,8]. Several active compounds from *R. glutinosa*, including iridoids, saccharides, and phenylethanoid glycosides (PhGs) have been discovered by pharmacological research [1].

**Figure 1:**
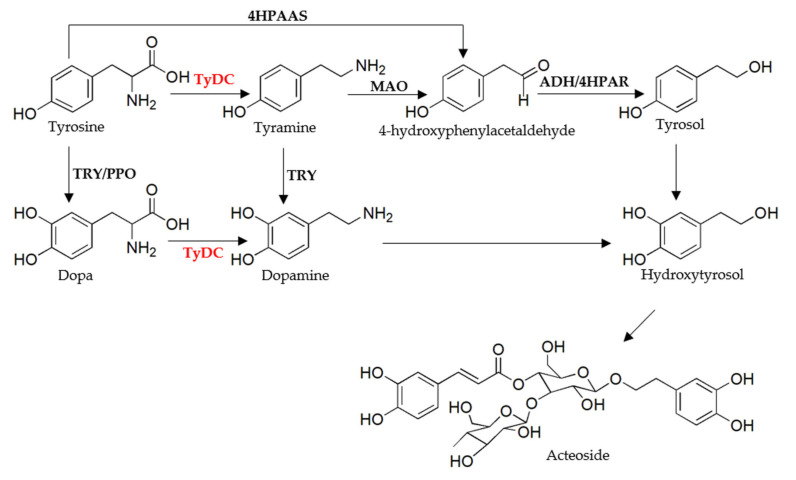
Proposed biosynthetic pathway of acteoside in *R. glutinosa* [15,16,17,18,19]. The enzyme identified in this study was highlighted in red. ADH, alcohol dehydrogenase; 4HPAAS, 4-hydroxyphenylacetaldehyde synthase; 4HPAR, 4-hydroxyphenylacetaldehyde reductase; MAO, monoamine oxidase; PPO, polyphenol oxidase; TRY, tyrosinase; TyDC, tyrosine decarboxylase.

Acteoside, an important bioactive compound of PhGs, accumulated in the leaves and tuberous roots of *R. glutinosa*. Acteoside drew attention due to its various biological activities, such as anti-inflammatory, antioxidant, anti-tumor, and neuroprotective properties [9]. Pharmacological research revealed acteoside could reduce mucosal tissue damage by inhibiting the oxidation of burst activity in inflammatory bowel disease treatment [10]. Wang’s study showed acteoside protects human neuroblastoma SH-SY5Y cells against β-amyloid-induced cell injury [11]. Acteoside also could inhibit cytokine production and NF-κB activation to ameliorate acute lung injury induced by lipopolysaccharide [12]. In addition, acteoside had positive effects against melanoma and hepatocellular carcinoma [13,14]. Although acteoside displayed a broad range of activities, its production mechanism in the plant was unclear. Acteoside was generated from the precursor phenylalanine and tyrosine which were produced via the shikimate pathway. Structurally, acteoside consisted of caffeoyl moiety and hydroxytyrosol, which were attached to a β-glucopyranose through a glycosidic bond [15]. The production of caffeoyl moiety was well studied, and feeding experiments revealed that l-phenylalanine is converted to the caffeoyl group via several intermediates, including cinnamic acid, coumaric acid, caffeic acid, and caffeoyl CoA under series enzymes [15]; whereas the hydroxytyrosol group might be generated by alternative pathways. Feeding experiments from *Syringa vulgaris* revealed tyrosine and tyramine can be efficiently converted into acteoside [16]. On the other hand, tyrosine could be transferred to acteoside via dopa and dopamine. Saimaru’s studies about *Olea europaea* showed that dopa produced from tyrosine is the main precursor for generating acteoside; dopa is converted to dopamine by dopa decarboxylase and then oxidated to aldehyde, reduced to hydroxytyrosol, and glycosylated to form acteoside in order [17]. In addition, the study about *Rhodiola* revealed that tyrosine can be converted to form 4-hydroxy-phenylacetaldehyde directly without the intermediate tyramine [18] and further reduced to tyrosol, which may be used to generate acteoside via hydroxytyrosol. The proposed biosynthesis steps of the hydroxytyrosol group and related enzymes are summarized in Figure 1 [15,16,17,18,19]. Although the hypothesized biosynthetic steps of acteoside seem reasonable, to date, enzymes or their corresponding genes involved in this pathway have not been characterized by enzymatic experiments. The biosynthetic pathway of acteoside with key genes remains to be studied.

Tyrosine decarboxylase (TyDC) from plants supported the biosynthesis of the PhGs and alkaloids by catalyzing the decarboxylation of tyrosine and dopa [20]. As shown in Figure 1, alternative pathways for acteoside accumulation might be available and TyDC played a vital role in the two biosynthetic pathways of acteoside. TyDC might participate in the formation of acteoside by producing tyramine and dopamine, and the enzymatic efficiency of TyDC determined the metabolite channeling at both branches. Thus, as a starting point to investigate the biosynthetic pathways of acteoside in *R. glutinosa,* the TyDC (denoted RgTyDC2) was isolated and characterized by biochemical assays. The role of RgTyDC2 in acteoside biosynthesis was explored by analyzing the *RgTyDC2* expression level and acteoside abundance in *R. glutinosa*. The findings here will aid understanding of the acteoside production in *R. glutinosa*.

## 2. Results

### 2.1. The Identification of Candidate Genes in the Biosynthesis of Acteoside

Based on the proposed biosynthesis pathway of acteoside in Figure 1, 181 sequences encoding 5 enzymes in this pathway were identified from the *R. glutinosa* transcriptome based on functional annotation, including 6 for TyDC, 13 for MAO, 138 for ADH, 12 for TRY, and 12 for PPO; the information of these sequences is shown in Table S1.

### 2.2. Identification and Sequence Analysis of R. glutinosa TyDC Genes

**Figure 2:**
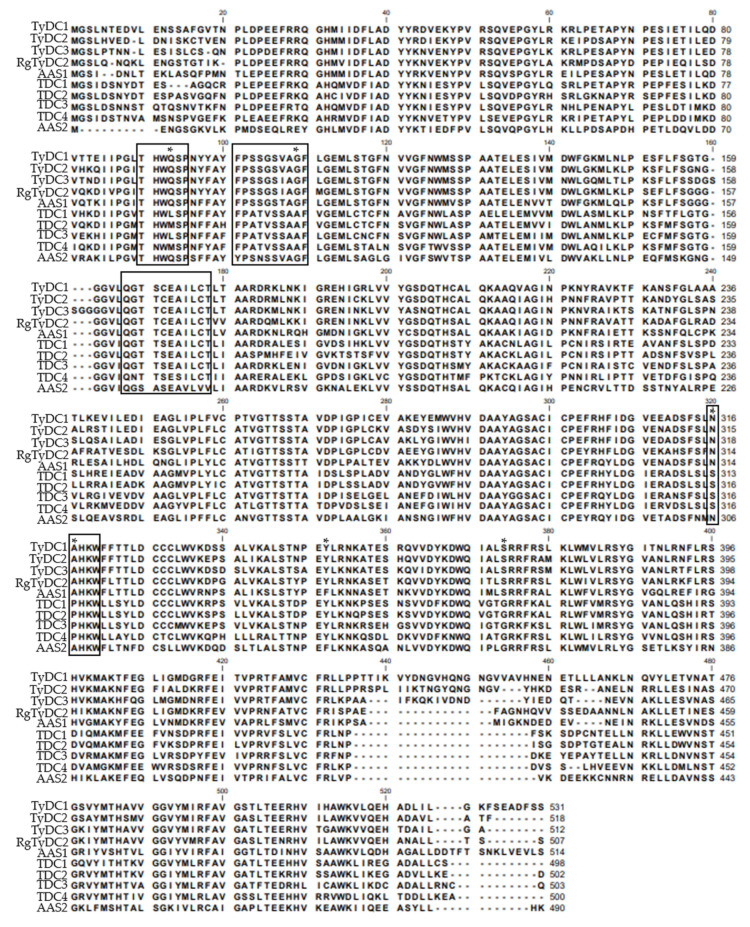
Sequence alignment of AAADs and RgTyDC2. Multiple sequence alignment was done by the CLC Sequence Viewer 6.8 program. Conserved motifs in TyDCs were indicated with boxes. Typical residues that dictated enzyme activities of AAADs were labeled with asterisks. TyDC1, AAC61843.1, *Papaver somniferum*; TyDC2, AAG60665.1, *Thalictrum flavum* subsp. glaucum; TyDC3, AAC61842.1, *Papaver somniferum*; TDC1, AAB39709.1, *Camptotheca acuminata*; TDC2, AAB39708.1, *Camptotheca acuminata*; TDC3, ACN62127.1, *Capsicum annuum*; TDC4, P17770.1, *Catharanthus roseus*; AAS1, Q06086.1, *Petroselinum crispum*; AAS2, NP_849999.1, *Arabidopsis thaliana*.

**Figure 3:**
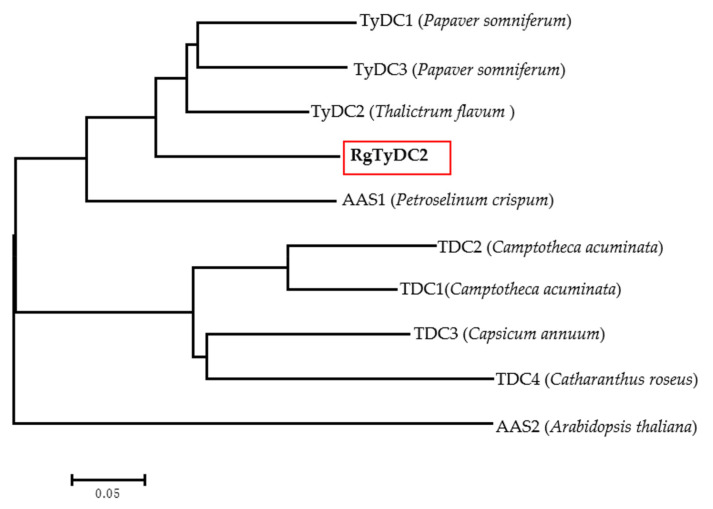
Phylogenetic analysis of RgTyDC2 with other known AAAD sequences. The amino acid sequence alignment was conducted using the Clustal X version 2 program. The tree was constructed with the Neighbor-Joining method of MEGA 7.0. The scale bar represents 0.05 amino acid substitutions per site. Accession numbers of AAAD sequences are provided in Figure 2.

Based on functional annotation, 6 sequences (Cluster-11149.35773, Cluster-11149.23630, Cluster-11149.20716, Cluster-11149.26764, Cluster-11149.28833, and Cluster-16731.0) were identified as tyrosine/dopa decarboxylase candidate genes from the *R. glutinosa* transcriptome. Cluster-11149.26764 and Cluster-11149.28833 were assembled into one sequence. Considering the other tyrosine decarboxylase (RgTyDC, KU640395) was isolated from *R. glutinosa* Wen 85-5 cultivar in a previous study [21], the TyDC sequence here was named *RgTyDC2* and selected for further analysis. Other sequences were discarded due to the uncompleted open reading frame (ORF) and low expression level (Table S1). *RgTyDC2* was amplified from leaves of the *R. glutinosa* Beijing No.3 cultivar by polymerase chain reaction (PCR) with gene-specific primers, and the sequence of *RgTyDC2* was deposited in the NCBI database (GenBank accession number OL744234). The ORF of *RgTyDC2* was 1524 bp and encoded a 56.05 kDa protein (507 amino acids). As literature reported, TyDCs together with tryptophan decarboxylases (TDCs) and aromatic acetaldehyde synthases (AASs) encompassed the aromatic amino acid decarboxylase (AAAD) family which was responsible for discrete decarboxylation or decarboxylation-deamination reactions of different aromatic amino acids. TyDC, TDC, and AAS shared high sequence similarity, which made it difficult to predict their function just based on the primary sequence [20]. Previous studies explored active site residues dictating catalytic functions; a Tyr^347^ (according to *Thalictrum flavum* TyDC, TyDC2, GenBank: AAG60665.1) was highly conserved in all identified TyDCs and TDCs, while Phe^346^ (according to *Petroselinum crispum* AAS, AAS1, GenBank: Q06086.1) was present in AASs [22]. Ser^372^ (according to *Papaver somniferum* TyDC, TyDC3, GenBank: AAC61842.1) residue was strongly conserved in TyDCs but substituted with glycine in TDCs [23]. To predict the catalytic activity, RgTyDC2 was aligned with several experimentally characterized enzymes from the plant AAAD family. As shownin Figure 2, RgTyDC2 had the typical Tyr and Ser of TyDC class. In common with other TyDCs, RgTyDC2 contained several conserved domains, the THWQSP motif, the [F/Y][P/A]S[S/N][G/S/T]S[I/V/T]AGF motif, the QGT[T/A/S][C/S]EA[V/I]L[C/V][T/V] motif and the NAHKW motif, which were involved in the binding of substrate and PLP (pyridoxal-5′-phosphate) [24,25]. The phylogenetic analysis in Figure 3 further showed that RgTyDC2 falls into the TyDC group and is most closely related to the TyDC (AAG60665.1) from *Thalictrum fiavum* (76% amino acid identity).

### 2.3. Functional Characterization of RgTyDC2

**Figure 4:**
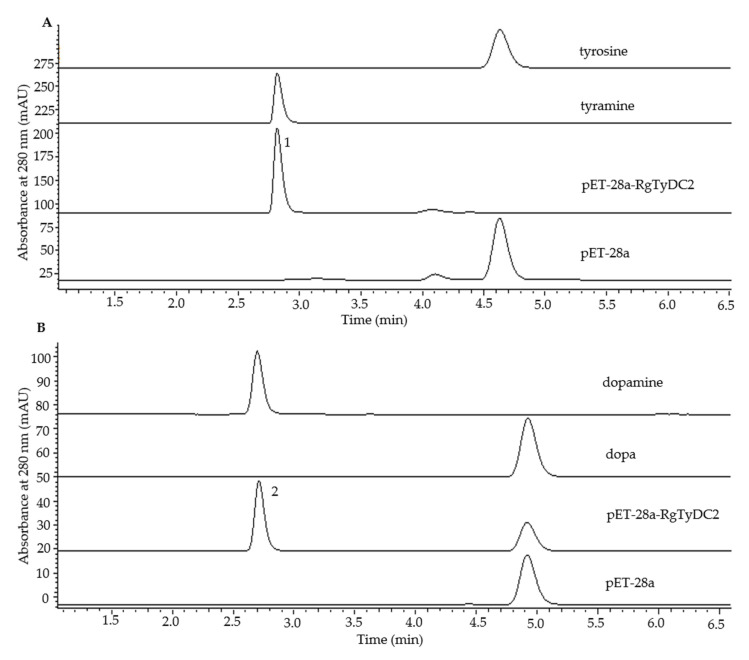
Functional characterization of the purified recombinant RgTyDC2 by in vitro enzyme assays. HPLC detection of the product of RgTyDC2 toward tyrosine and dopa is shown in (**A**) and (**B**), respectively. RgTyDC2 converted nearly all tyrosine to tyramine (peak 1) and part of dopa to dopamine (peak 2). These products were not detected in the control reactions which used protein from *E. coli* cells harboring the empty vector pET-28a.

**Figure 5:**
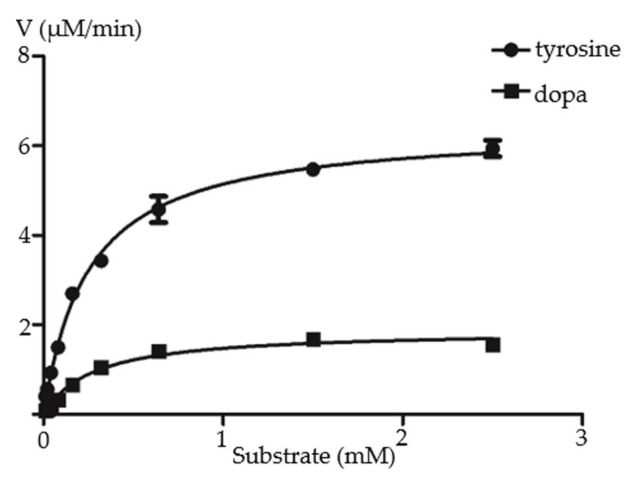
Kinetic characterization of RgTyDC2 against the substrate tyrosine and dopa. The kinetic values *K*_m_ and *V*_max_ of RgTyDC2 toward tyrosine were 249.7 ± 17.11 μM and 6.424 ± 0.1312 μM min^−1^, respectively. The *K*_m_ and *V*_max_ values when dopa was applied as the substrate were 273.8 ± 37.18 μM and 1.878 ± 0.0776 μM min^−1^, respectively.

To examine the biochemical activity of RgTyDC2, it was expressed in *E. coli* BL21(DE3) cells and purified using His tag. The recombined protein was assayed to verify its activity with the substrate tyrosine, dopa, tryptophan, and phenylalanine. HPLC detection of the enzymatic products showed that compared with the control reaction, the new compounds tyramine and dopamine are produced by RgTyDC2 protein when using tyrosine and dopa as substrate (Figure 4), respectively, whereas no product is detected when using tryptophan and phenylalanine as substrate. The enzyme kinetic properties of RgTyDC2 toward tyrosine and dopa were further measured. The *k*_cat_ and *K*_m_ values are shown in Table 1 and the progress curve is shown in Figure 5. The catalytic efficiency (*k_cat_*/*K_m_*) of RgTyDC2 was about 4.4-fold higher for tyrosine than for dopa.

**Table 1:** Kinetic parameters for RgTyDC2 toward tyrosine and dopa.

Substrate	*k*_cat_ (s^−1^)	*K*_m_ (μM)	*k*_cat_/*K*_m_ (s^−1^M^−1^)
tyrosine	0.507 ± 0.0104	249.7 ± 17.11	2.032 × 10^3^
dopa	0.126 ± 0.0052	273.8 ± 37.18	4.602 × 10^2^

### 2.4. Gene Expression Data of RgTyDC2 Was Consistent with the Accumulation Pattern of Acteoside in R. glutinosa

**Figure 6:**
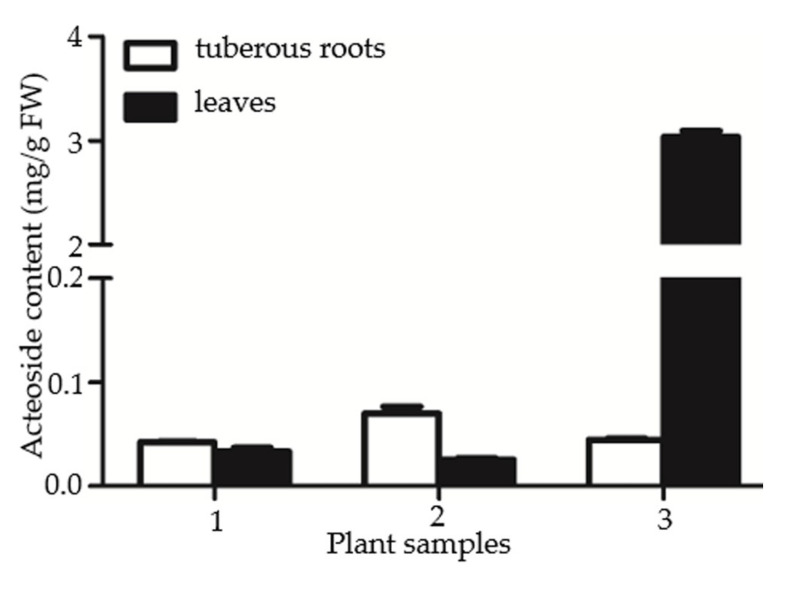
The accumulation pattern of acteoside in leaves and tuberous roots of three *R. glutinosa* plant samples. Error bars represent the standard errors of the means calculated from three independent technical replicates.

**Figure 7:**
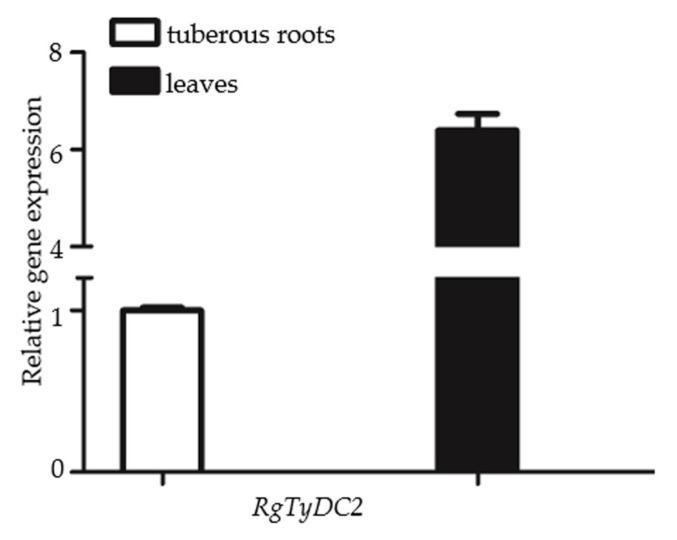
The transcript level of *RgTyDC2* in leaves and tuberous roots of *R. glutinosa*. QRT-PCR was applied to analyze *RgTyDC2* transcripts. The expression level was normalized to that of an *R. glutinosa* gene *TIP41*. RNA was extracted from the plant, which showed higher acteoside content in leaves than in tuberous roots. Error bars represent the mean± SD from three technical replicates.

To investigate the role of RgTyDC2 in the biosynthesis of acteoside in *R. glutinosa*, the correlation between acteoside content and *RgTyDC2* gene expression was evaluated. The production of acteoside in leaves and tuberous roots of *R. glutinosa* was detected by HPLC. Among three independent biological experiments, one plant sample showed higher acteoside abundance in leaves than in tuberous roots, while the others showed similar concentrations between the two organs (Figure 6). Additionally, then the one *R. glutinosa* plant sample, in which acteoside accumulation displayed leaves specific, was just chosen for further gene expression analysis; the higher mRNA level of *RgTyDC2* was detected in leaves than in tuberous roots (Figure 7), matching the distribution property of acteoside of *R. glutinosa*.

## 3. Discussion

*R. glutinosa* was an important Chinese medicinal plant and acteoside was one of the active metabolites isolated from *R. glutinosa.* Acteoside showed remarkable biological activities, including anti-inflammatory, anti-tumor and antioxidant effects [15]. As shown in Figure 1, acteoside was produced from precursor phenylalanine and tyrosine. It is well known that phenylalanine was converted to form the caffeoyl moiety of acteoside through the cinnamate pathway. The biosynthesis of its hydroxytyrosol part needed to be fully elucidated. Isotope-labeled precursor feeding experiments showed that the hydroxytyrosol can be synthesized from tyrosine via tyramine and/or via dopa, or via 4-hydroxy-phenylacetaldehyde [15,16,17,18], suggesting the production of acteoside may be accomplished through alternative pathways in the plant. As shown in Figure 1, the conversion of tyrosine was the first branch point for the biosynthesis of acteoside and TyDC was the committed enzyme in the pathway. Furthermore, to elucidate the molecular biosynthesis mechanism of acteoside, we constructed transcriptome from leaves and tuberous roots of *R. glutinosa* using the Illumina NovaSeq 6000 platform, and 181 unigenes corresponding to 5 enzymes in the acteoside biosynthetic pathway were identified. The number of putative genes involved in acteoside production was more than those found in the *R. glutinosa* transcriptome in previous studies [19,26], indicating the transcriptome library in the current study is sufficient to discover genes in acteoside biosynthesis. The enzymes in Figure 1 were all identified, except 4HPAAS and 4HPAR, which catalyzed the decarboxylation-deamination of tyrosine and aldehyde reduction reaction in sequence to produce tyrosol, demonstrating tyrosine could not be converted to 4-hydroxy-phenylacetaldehyde directly. Thus, we concluded that the tyrosine is converted to dopa or tyramine firstly in the biosynthesis of acteoside in *R. glutinosa*, which was consistent with findings from previous studies that tyramine and dopa participate in the formation of acteoside [16,17].

TyDC catalyzed the decarboxylation of aromatic amino acids with phenol side chains, such as tyrosine. In this study, one sequence was identified as TyDC from the *R. glutinosa* transcriptome and named *RgTyDC2,* which was isolated from the *R. glutinosa* Beijing No.3 cultivar using the PCR method. The other TyDC named RgTyDC was already cloned from the *R. glutinosa* Wen 85-5 cultivar, but without enzymatic experiment for functional characterization in Wang’s study [21]. The amino acid sequence of RgTyDC2 shared 87% identity with the RgTyDC, indicating they were different sequences. Further, it was supposed that tyrosine decarboxylase may be multiple members in *R. glutinosa* and different in various cultivars. As indicated by the result of primary sequence alignment and phylogenetic analysis, RgTyDC2 contained typical conserved motifs of TyDCs (Figure 2) and was most closely related to the TyDC (AAG60665.1) from *Thalictrum flavum* (Figure 3), suggesting RgTyDC2 belonged to the TyDC class. However, all TyDCs from plants showed activity for tyrosine and dopa, but the relative enzyme activity toward these two substrates was different among various plants. Functional characterization by in vitro biochemical assay revealed that like the reported TyDCs from *Papaver somniferum* and *Pertoselinum crispum* [20], RgTyDC2 converts tyrosine and dopa to tyramine and dopamine, respectively. For the enzyme kinetic parameters (Table 1), the *K*m value of RgTyDC2 toward tyrosine was similar to TyDCs from *Lycoris radiata* and *Thalictrum rugosum* [20,27], while it showed much more differences with that of TyDC from *Rhodiola sachalinensis* [28]. RgTyDC2 has a higher substrate preference for tyrosine (with low *K*m and high *k*cat/*K*m values). The catalytic efficiency (*k*cat/*K*m) of RgTyDC2 was about 4.4 times higher for tyrosine than for dopa, and the V_max_ of RgTyDC2 for tyrosine was about 3.4-fold higher than those for dopa, suggesting that tyrosine was the better substrate for RgTyDC2. Similarly, a higher affinity toward tyrosine was observed in TyDCs from *Rhodiola sachalinensis* and *Pertoselinum crispum*, whereas TyDCs from *Hordeum vulgare*, *Sanguinaria canadensis*, and *Cytisus scoparius* showed a preference for dopa [20]. Based on the enzymatic data of RgTyDC2, we concluded that the pathway from tyrosine to acteoside via tyramine is the main pathway for producing acteoside in *R. glutinosa*; in addition, the pathway from tyrosine to dopa was also available in the *R. glutinosa*.

*R. glutinosa* Beijing No.3 cultivar was selected as our plant material. From three biological replicates, only one *R. glutinosa* plant sample showed significantly higher acteoside content in leaves than that in tuberous roots, revealing the accumulation pattern of acteoside in leaves and tuberous roots of *R. glutinosa* Beijing No.3 cultivar was not regular among different plant samples. This result was different from Wang’s studies showing the acteoside content in leaves is often ten times more than that in roots of *R. glutinosa* Wen 85-5 and QH cultivars [19], which might result from species or growth condition difference. The one *R. glutinosa* plant which produced more acteoside in leaves was chosen for further gene expression analysis and *RgTyDC2* expressed higher in leaves compared with tuberous roots (Figure 7), suggesting under the condition of this study, the *RgTyDC2* expression data was consistent with the acteoside accumulation pattern in *R. glutinosa*. Thus, it was reasonable to assume the possible roles of RgTyDC2 in the formation of acteoside in *R. glutinosa*. However, understanding the physiological roles of RgTyDC2 in vivo could be achieved by overexpression and knock-down application in the *R. glutinosa* plant.

## 4. Materials and Methods

### 4.1. Plant Materials and Chemicals

*R. glutinosa* plants of Beijing No.3 cultivar were grown in an incubator illuminated with white fluorescent light (4000 lx; 16 h light period/day) at 23 ℃. The leaves and tuberous roots were harvested 180 days after sprouting. Acteoside, l-tyrosine, tyramine, l-tryptophan, tryptamine, l-dopa, dopamine, l-phenylalanine, phenethylamine, and pyridoxal 1-phosphate were purchased from Shanghai Source Leaf Biological Technology Company (Shanghai, China, http://www.shyuanye.com). Methanol was from Thermo Fisher Scientific Inc. (Waltham, MA, USA). HCl was from the Shanghai Hushi laboratory equipment company. Unless specified otherwise, all enzymes were purchased from Takara Company (Dalian, China).

### 4.2. Identification and Cloning of the Tyrosine Decarboxylase from R. glutinosa

*R. glutinosa* transcriptome database derived from the leaves and tuberous roots was recently constructed by our group (BIG Data Center, accession No. CRA005581). *R. glutinosa* tyrosine/dopa decarboxylase genes were screened based on functional annotation in the transcriptome database. Multiple sequence alignment was conducted using the CLC Sequence Viewer 6.8 program and the phylogenetic tree was generated by the neighbor-joining method of the MEGA 7.0, using 1000 bootstrap replications.

### 4.3. Heterologous Expression of RgTyDC2

For the functional characterization, primers 1 and 2 (Table S2) were designed to amplify the full-length coding region fragment of *RgTyDC2* by TransStart FastPfu DNA polymerase (TransGen Biotech, Beijing, China). *RgTyDC2* were subcloned into the plasmid pET-28a at the EcoR I/Not I site, giving the pET-28a-*RgTyDC2*. The construct pET-28a-*RgTyDC2* was transferred into *E. coli* BL21 (DE3) cells. The expression of *RgTyDC2* was induced with 1 mM isopropyl-β-d-thiogalactopyranoside (IPTG), which was cultivated at 16 ℃, 180 rpm for 14 h. After induction, transgenic *E. coli* cells were harvested by centrifugation at 8000× *g* for 5 min, resuspended in the chilled lysis buffer (20 mM sodium phosphate, 300 mM sodium chloride with 10 mM imidazole; pH 7.4) and disrupted by ultrasonication. The crude protein extracts were collected by centrifugation at 12,000× *g* for 10 min at 4 ℃ and then loaded onto a column packed with HisPur Ni-NTA resin to purify RgTyDC2 according to the manufacturer’s instructions. The purified recombinant proteins were desalted into 50 mM Tris-HCl (pH 7.2) buffer with 5% glycerol. The purity of recombinant RgTyDC2 was assessed by SDS-PAGE and its concentration was determined by the Bio-Rad protein assay.

### 4.4. Enzyme Assays

The standard in vitro enzymatic activity assays were performed in 250 μL of 50 mM Tris-HCl buffer (pH 7.2) containing 25 μM pyridoxal 1-phosphate (PLP), 1 mM aromatic amino acid substrate (l-tyrosine, l-tryptophan, l-dopa, l-phenylalanine), 1.0 mM EDTA, and 10 μg of isolated protein. After incubation at 30 ℃ for 2 h, the reaction mixture was collected for high-performance liquid chromatography (HPLC) analysis.

For kinetic analysis of RgTyDC2, a 250 μL reaction mixture including 50 mM Tris-HCl (pH 7.2), 25 μM PLP, 1.0 mM EDTA and 10 μM to 2.5 mM of substrates including tyrosine and dopa, were performed at 30 ℃ for 50 min before HPLC analysis. The kinetic values were determined by hyperbolic regression analysis using the GraphPad Prism 5 program.

### 4.5. Determination of Acteoside from Plant Materials

To explore the organ specificity of acteoside accumulation in *R. glutinosa*, three unfolded leaves were mixed and ground to powder in liquid nitrogen, which was used as the leaves material of this plant. Tuberous roots were also ground to powder. For the acteoside extraction, 100 mg of fresh plant materials (leaves and tuberous roots) were extracted twice with 500 μL of methanol for 30 min by ultrasonic extraction at room temperature. The supernatants were combined, then dried by vacuum and re-dissolved in 800 μL of 50% methanol, which was filtered through the 0.22 μm microfilter prior to HPLC detection.

### 4.6. HPLC Analysis

HPLC detection was conducted using an LC-16AT system equipped with an Inertsil ODS-SP reverse phase column (250 × 4.6 mm, 5 μm) (Shimadzu, Kyoto, Japan) and mobile phase containing solvent A (water) and solvent B (methanol). To analyze acteoside from plant materials, the samples were eluted with 40% methanol at a flow rate of 0.6 mL min^−1^. The column temperature was 25 ℃ and absorbance was 330 nm. Quantification of acteoside was determined from three biological replicates using the standard curves method.

For detecting the products from RgTyDC2 in vitro assay, 20% methanol was used as the mobile phase at a flow rate of 0.6 mL min^−1^. The detection wavelength was set at 280 nm and the column temperature was 30 ℃. The compound was confirmed by comparing their retention times with authentic standards.

### 4.7. Real-Time PCR

A quantitative reverse transcriptional polymerase chain reaction (QRT-PCR) was used to analyze gene expression levels. Total RNA was isolated from the plant sample which produced more acteoside in leaves than in tuberous roots using the EASYspin plus Plant RNA isolation kit (Aidlab Biotech, Beijing, China). The RNAwas treated with DNase I and then reverse transcribed to first-strand cDNA using Revert Aid reverse transcriptase (Thermo Fisher Scientific, Waltham, MA, USA). QRT-PCR was performed with the LightCycler 480 system (Roche, Mannheim, Germany) using TransStart Top Green qPCR SuperMix (TransGen Biotech, Beijing, China) in three biological replicates with three technical replicates. *TIP41* gene, which was stably expressed in different organs of *R. glutinosa*, was used as a reference gene [29] and amplified with primers 3 and 4. Primers 5 and 6 were designed to amplify *RgTyDC2* for the QRT-PCR (Table S2). The thermal cycling conditions were set as follows: 95 ℃ for 5 min, then 40 cycles of 95 ℃ for 10 s, 56 ℃ for 10 s and 72 ℃ for 10 s.

## 5. Conclusions

In this study, the cDNA encoding a novel tyrosine decarboxylase (*RgTyDC2*) was isolated from *R. glutinosa*. RgTyDC2 was responsible for the decarboxylation of tyrosine and dopa and exhibited a preference for tyrosine. The gene expression of RgTyDC2 matched the abundance of acteoside in *R. glutinosa*.

## Data Availability

The transcriptome data has been deposited in public accessible Genome Sequence Archive (GSA) database in the BIG Data Center under the accession number CRA005581.

## Supplementary Materials

The following supporting information is available online, Table S1: Putative enzymes in acteoside biosynthesis pathway; Table S2: Primers used in this study.
