# Supplementary material for: Identification and Functional Characterization of Tyrosine Decarboxylase from Rehmannia glutinosa
Source: Molecules. 2022 Mar 1;27(5):1634. doi: 10.3390/molecules27051634 (PMC8912026; doi:10.3390/molecules27051634)
Supplement: Supplementary file 1 [file molecules-27-01634-s001.zip › Table S2 Primers used in this study.pdf]

Table S2. Primers used in this study

Number of the primers	Names of primers	Sequences of the primers (5′-3′)	Descriptions
Primer 1	RgTyDC2-FP	ATGGGCAGCCTTCAGAATCA	For amplifying RgTyDC2
Primer 2	RgTyDC2-RP	TCACGAACTGGTTAGCAGAGCA	For amplifying RgTyDC2
Primer 3	TIP41-RT-FP	ATGCGGCTGGAAGCAGGAAG	For amplifying TIP41 transcripts in QRT-PCR
Primer 4	TIP41-RT-RP	GCCAACGCGACCATATCAA	For amplifying TIP41 transcripts in QRT-PCR
Primer 5	RgTyDC2-RT-FP	ATCAGACCCATTCCGCATTA	For amplifying RgTyDC2 transcripts in QRT-PCR
Primer 6	RgTyDC2-RT-RP	CGCAGAGGAACAGAGGCAC	For amplifying RgTyDC2 transcripts in QRT-PCR
